# Very Long-Term Treatment with a *Lactobacillus* Probiotic Preparation, *Lactobacillus Casei* Strain Shirota, Suppresses Weight Loss in the Elderly

**DOI:** 10.3390/nu12061599

**Published:** 2020-05-29

**Authors:** Hideki Ishikawa, Michihiro Mutoh, Kenichi Yoshimura, Gen Fujii, Tomiyo Nakamura, Tatsuya Takeshita, Keiji Wakabayashi, Toshiyuki Sakai

**Affiliations:** 1 Department of Molecular-Targeting Prevention, Kyoto Prefectural University of Medicine, Kyoto 602-0841, Japan; cancer@gol.com; 2 Ishikawa Gastroenterology Clinic, Osaka 541-0042, Japan; 3 Epidemiology and Prevention Group, Research Center for Cancer Prevention and Screening, National Cancer Center, Tokyo 104-0045, Japan; 4 Center for Integrated Medical Research, Hiroshima University Hospital, Hiroshima 734-8551, Japan; keyoshim@hiroshima-u.ac.jp; 5 Central Radioisotope Division, National Cancer Center Research Institute, Tokyo 104-0045, Japan; gfujii@ncc.go.jp; 6 Department of Food Sciences and Human Nutrition, Ryukoku University, Shiga 520-2194, Japan; tomiyo@agr.ryukoku.ac.jp; 7 Department of Public Health, Wakayama Medical University School of Medicine, Wakayama 641-8509, Japan; ttakeshi@wakayama-med.ac.jp; 8 Graduate Division of Nutritional and Environmental Sciences, University of Shizuoka, Shizuoka 422-8526, Japan; kwakabayashi@u-shizuoka-ken.ac.jp; 9 Department of Drug Discovery Medicine, Kyoto Prefectural University of Medicine, Kyoto 602-8566, Japan; tsakai@koto.kpu-m.ac.jp

**Keywords:** BLP, *Lactobacillus*, long-term treatment, weight loss

## Abstract

Weight loss, often observed in the elderly, is associated with increased risks of various diseases. No large and long-term human study has been conducted to demonstrate the health maintenance-related effects of lactic acid bacteria preparations. To reveal the potential benefit of long-term lactic acid, the effects of bacteria-based probiotics for health maintenance were examined. This observational study included the participants from a previous clinical study designed to evaluate the effects of wheat bran biscuits or *Lactobacillus* preparation, 3 g/day biolactis powder (BLP), in preventing colorectal tumor. The participants were provided an option to continue treatment with BLP on an outpatient basis after completion of the study. The 380 patients who completed the study were contacted and asked to participate in the present study and those who consented were surveyed for cancer incidence, treatment compliance, lifestyle, weight, and other variables. Informed consent was obtained from 237 of the 380 (62.4%) patients. The mean follow-up period was 7913 days (21.7 years). Cancer developed in 24 of 128 (18.8%) patients in the BLP extension group and 24 of 109 (22.0%) patients in the non-BLP extension group (risk ratio 0.88 [95% confidence interval 0.53–1.47]). Although no significant difference was observed, the cumulative cancer incidence rose at a slightly lower rate in the BLP extension group. Both groups showed a significant weight decrease over the course of 20 years, although the decrease in the BLP extension group was only 1.4 kg, compared with 2.8 kg in the non-BLP extension group. Very long-term treatment with a *Lactobacillus* probiotic preparation suppressed weight loss in the elderly.

## 1. Introduction

Reduced nutritional intake due to reduced appetite in the elderly is associated with various problems, including muscle weakness, osteoporosis, anemia, impaired immune function and depressive symptoms. Weight loss is used as a measure of reduced nutritional intake. Emerging evidence has suggested associations between intestinal bacteria and various diseases [1,2]. Although studies have suggested the potential benefit of lactic acid bacteria-based probiotics for health maintenance [3,4], no large-scale human study has been conducted to demonstrate the health maintenance-related effects of long-term treatment with lactic acid bacteria probiotic preparations.

As part of the “10-year Strategy for Cancer Control” implemented by the Ministry of Health, Labour and Welfare (MHLW), we conducted a clinical study to evaluate the efficacy of wheat bran and a *Lactobacillus* probiotic preparation biolactis powder (BLP) containing *Lactobacillus casei* strain Shirota for preventing colorectal cancer [5] and found that treatment with BLP significantly reduced the incidence of adenoma graded as moderate atypia or worse, a precancerous lesion of colorectal cancer. In the patients treated with wheat bran, the 4-year incidence of colorectal tumors tended to increase, with a relative risk of 1.31 (95% confidence interval (CI): 0.87–1.97), and that of tumors measuring ≥3 mm significantly increased, with a relative risk of 1.57 (95% CI: 1.04–2.37). In contrast, in those treated with BLP, the 4-year incidence of colorectal tumors slightly decreased, with a relative risk of 0.85 (95% CI: 0.56–1.27), and that of tumors graded as moderate atypia or worse significantly decreased, with a relative risk of 0.65 (95% CI: 0.43–0.98). There were no adverts events according to the BLP intake. The study participants were informed of these results and provided an option to continue treatment with BLP on a prescription basis after completing the study. After more than 20 years had passed since commencement of the study, we contacted the study participants again to ask about their clinical courses since their study participation and also planned and conducted a new study to further follow them.

The objective of this study was to evaluate the effects of long-term treatment with BLP by comparing cancer incidence, morbidity, concomitant medications, exercise habit, weight and other variables between the participants who continued the treatment and those who did not.

## 2. Materials and Methods

### 2.1. Patients

This study included the participants in a previous clinical study designed to evaluate the effects of wheat bran and BLP in preventing colorectal cancer (wheat bran biscuit (WBB)/BLP study) (5). Briefly, the WBB/BLP study was conducted at a single, large-scale medical institution in Osaka prefecture, Japan (Osaka Medical Center for Cancer and Cardiovascular Diseases) between 1993 and 1997 and included male and female patients between 40 and 65 years of age who had two or more colorectal tumors (adenoma or early cancer) and had all of their tumors removed endoscopically within 3 months before entry.

The participants were randomly assigned to receive one of the following 4 treatments: WBB alone (group A), BLP alone (group B), WBB plus BLP (group C) and no treatment (group D). All participants were asked about their dietary habits and received dietary instructions. The intervention was performed for 4 years. Colonoscopy was performed after 2 and 4 years of treatment and any newly detected colorectal polyps were removed and examined histologically. This study was supported by a grant-in-aid from the “10-year Strategy for Cancer Control” project of the MHLW (Kakizoe/Wakabayashi group).

Of the 470 eligible patients, 60 refused to participate, 12 were excluded and the remaining 398 participated in the study. Eighteen (4.5%) patients failed to complete the 4-year study period. The reasons for withdrawal included death (lung cancer, cerebral hemorrhage; *n* = 2), serious diseases (peritonitis from acute appendicitis, subarachnoid hemorrhage, knee arthritis, cardiac infarction, gastric cancer; *n* = 5), job relocation (*n* = 1), patient’s wish to undergo barium enema examination (*n* = 2) and patient’s wish to discontinue the study (*n* = 8). The remaining 380 patients were included in the analysis.

During the 4 years of the intervention study, approximately half of the patients were assigned to receive BLP. After the study period, patients who participated in the intervention study were asked whether they would continue and take a drug the same as BLP, regardless of whether they had been in the BLP group or not. Of note, multivariate adjusted ORs for occurrence of tumors were 0.76 (0.50–1.15) in the BLP treated group compared to the control group. However, tumor incidence? size? did not reduce in the group consuming the wheat bran biscuits. In patients who desired to continue, they received ‘Biolactis’, which contains the same ingredients as BLP, at a dose of 3 g/day (divided into 3 doses after meals) on an outpatient basis. In Japan, ‘Biolactis’ can be prescribed on medical insurance. Those who wished to be followed-up by colonoscopy underwent this procedure every 2–3 years after completing the study.

### 2.2. Study Design and Intervention

Along with the transfer of the attending physician (Hideki Ishikawa), the study site in which the participants received outpatient treatment also changed to Osaka Central Hospital in 2002 and to the Ishikawa Gastroenterology Clinic in 2008.

In 2016, we contacted the 380 patients who completed the WBB/BLP study and asked them to consent to the provision of information about their clinical courses during the time between participation in the WBB/BLP study and December 2018. Then, we designed a long-term follow-up study.

Patients receiving outpatient treatment at Ishikawa Gastroenterology Clinic were interviewed and provided written informed consent during regular visits. Those who discontinued their outpatient visits were contacted by telephone, mail, or other means to obtain consent. For those who had died, consent was obtained from family members. Those from whom could not be obtained informed consent, due to being lost to follow-up, were excluded.

We collected the participants’ medical information and other relevant information accumulated between the time of their participation in the WBB/BLP study and December 2018. The participants’ blood testing and colonoscopy data generated during their participation in the 4-year WBB/BLP study was collected from their medical records. Their height/weight measurements at the time point of participated in the WBB/BLP study was obtained by the hospital staff. Their compliance with treatment, illness, height/weight and dietary information after completion of the WBB/BLP study was collected using a self-completion questionnaire. Those taking BLP at the time of the questionnaire survey were defined as the BLP extension group.

### 2.3. Ethics

The study was conducted in accordance with the guiding principles of the Declaration of Helsinki and was approved by the ethics committee at Kyoto Prefectural University of Medicine (approval No. UMIN000025389). Participants gave oral and written informed consent before enrollment. Clinical Trial Registry number: No. UMIN000025389. URL of registration: https://upload.umin.ac.jp/cgi-open-bin/ctr/index.cgi

### 2.4. Statistical Analysis

Statistical analyses were performed to compare the BLP extension and the non-BLP extension groups. The unpaired or paired t-test was used for comparisons of continuous variables, the chi-square test for comparisons using a contingency table, and the log rank test for comparisons of cancer incidence. The cancer incidence after participation in the WBB/BLP study was defined as the incidence after completion of the 4-year intervention in the WBB/BLP study. Statistical processing was performed using JMP^®^ software (Ver. 14.0.0). Differences were considered statistically significant at a value of *p* < 0.05.

## 3. Results

### 3.1. Patients

**Figure 1:**
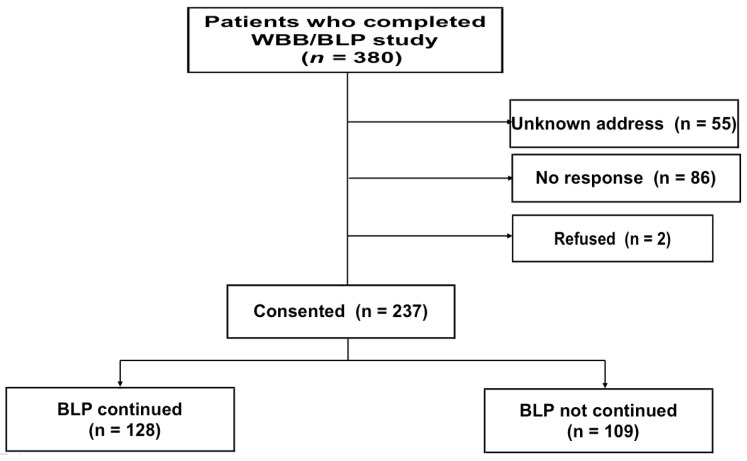
Flowchart of study entry.

Of the 380 eligible patients, 55 could not be contacted due to unknown residential addresses, 86 did not respond to the letters sent to known residential addresses, and 2 refused to participate (Figure 1). Thus, consent was obtained from the remaining 237 patients (62.4%; 218 consents from patients and 19 consents from family members). Eighteen consents were obtained from the deceased patients’ family members who received the letter and 1 consent from the family of a patient who was too ill to be able to express his/her will.

The mean interval from the day of consent to participation in the WBB/BLP study until the day of completion of the questionnaire was 7913 days (21.7 years; range 6789 to 9157 days, with a standard deviation of 600 days).

The baseline characteristics of the consenting patients are summarized in Table 1. There were 199 males (84.0%), the percentage being almost the same as that in the WBB/BLP study (84%). Of the consenting patients, 128 (54.2%) had been treated continuously with BLP at the time of consent. No significant difference was found between those with and without ongoing treatment with BLP with respect to sex, age, or height/weight at participation in the WBB/BLP study. Two of the participants in the WBB/BLP study lacked height/weight data.

### 3.2. Cancer Development

**Figure 2:**
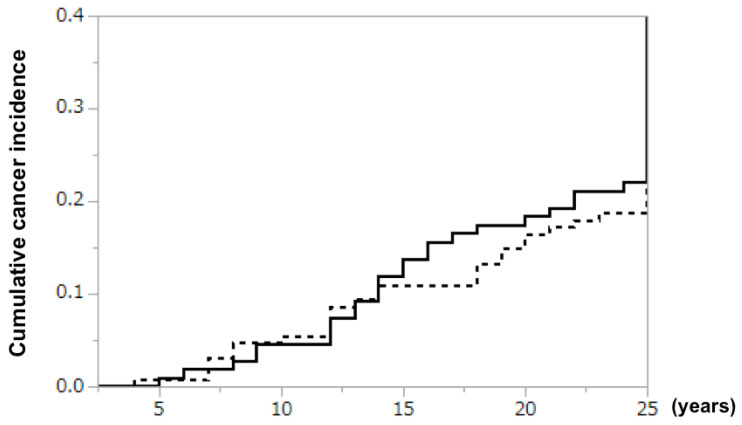
Cumulative cancer incidence in BLP extension group and non-BLP extension group. Dotted line: Treated with Biolactis. Solid line: Not treated with Biolactis.

Cancer developed in 24 of 128 (18.8%) patients in the BLP extension group and 24 of 109 (22.0%) patients in the non-BLP extension group (risk ratio 0.88 [95% CI 0.53–1.47], log rank test; Figure 2). Although no significant difference was observed, the cumulative cancer incidence increased at a slightly lower rate in the BLP extension group from year 15 onward. Metachronous double cancer occurred in 4 (including 1 with triple cancer) and 6 patients in the BLP extension and non-BLP extension groups, respectively. The most common type of malignancy was gastric cancer in both groups, with similar incidences in the two groups (Table 2).

### 3.3. Body Weight Changes and Other Findings

When asked whether they were currently aware of any illness, 31 (24.2%) and 22 (19.4%) patients answered “No” in the BLP extension and non-BLP extension groups, respectively; thus, there were 1.25-fold more illness-free patients in the BLP extension group.

When asked whether they were prescribed any medications by their physicians at the time of completion of the questionnaire, 30 (23.4%) and 33 (30.3%) patients answered “No” in the BLP extension and non-BLP extension groups, respectively; thus, slightly fewer patients were not taking medications in the BLP extension group. The most common drug type was antihypertensive, followed by antithrombotic, antilipemic, antidiabetic and antiuricemic agents, with no significant differences between the two groups.

The number (percentage) of patients engaged in regular exercise was 71 (55.5%) in the BLP extension and 54 (50.0%) in the non-BLP extension group, with no significant difference.

The analysis of weight included participants with available before-and-after data: 124 from the BLP extension and 91 from the non-BLP extension group (Table 3). Although both groups showed a significant weight decrease over the course of 20 years, the decrease in the BLP extension group was only 1.4 kg, compared with 2.8 kg in the non-BLP extension group. Moreover, the decrease in body mass index (BMI) was significant in the BLP extension and insignificant in the non-BLP extension group.

No serious adverse reactions to BLP were documented in any patient.

## 4. Discussion

The results of the present study demonstrated that although the participants’ weights decreased substantially over the course of approximately 20 years, very long-term treatment with a *Lactobacillus* probiotic preparation reduced the magnitude of these weight decreases.

The mechanism by which long-term treatment with BLP suppressed weight loss remains unknown. Unlike lactic acid bacteria-containing beverages, BLP does not contain sugar or other major sources of calories. We speculate that lactic acid bacteria produce organic acids in the intestine and thereby convert dietary fibers into substances that can be used for energy production, such as short-chain fatty acids, and that these substances were used to produce energy and thereby suppressed weight loss [6,7,8]. Of note, other members of the endogenous microflora also ferment fiber to produce hort-chain fatty acids.

Weight loss in the elderly is associated with increased risk of “frailty (a condition of being vulnerable to stress)” [9]. Frailty in the elderly is known to be associated with reduced quality of life, as well as various morbidities. Since it is believed that many elderly people become frail before they require nursing care, preventing frailty is very important for health management of the elderly.

The fact that the BLP extension group only showed a mild weight decrease over more than 20 years suggests that the long-term use of BLP helps maintain resistance to frailty. Since elderly people are also prone to malnutrition due to reduced appetite, calories supplied by such means appear to be an important source of nutrition in the elderly.

A slight reduction in cumulative cancer incidence was noted after approximately 15 years of treatment with BLP. Because it takes time before a clinical diagnosis of cancer is made after its development, it is understandable that the cancer risk reduction effect became evident only after the relatively long treatment period of approximately 15 years. Many studies have suggested the potential of BLP for the prevention of cancer development [10,11,12].

While serious adverse events have been reported in association with the administration of lactic acid bacteria probiotic preparations [13] and stool transplantation [14], the observation from the present study that there were no reports of serious adverse reactions in a relatively large population of patients receiving long-term treatment with BLP demonstrates not only the short-term, but also the long-term safety of BLP. No serious side effects have ever been reported for BLP.

The present study has a number of advantages. First, the study included participants in the completed WBB/BLP study, making confirmed enrollment information of the participants and abundant medical information obtained during the previous study available to researchers. Second, a high re-consent rate was achieved because many patients continued outpatient visits to the office of the same attending physician. Lastly, more than 100 patients were continuously prescribed BLP, a quality controlled medicinal product, for more than 20 years and their compliance with this treatment could be followed through regular visits. It appears that patients rarely missed doses because they are paying for the BLP themselves.

The limitations of this study include a possible selection bias as most of the participants who did not respond to the request for study participation were likely to have died, although this would probably lead to underestimation of the efficacy of BLP, given that few patients died during ongoing treatment with BLP and the majority of the patients who discontinued outpatient visits were those not treated with BLP. In addition, it is estimated that about 7% of the people in Japan drink Shirota strain lactic acid bacteria-containing beverages daily. Therefore, even in the BLP non-administration group, about 7% may be ingesting the Shirota strain lactic acid bacterium, which may weaken/diminish the results of this study.

Furthermore, the data pertaining to recent weight, cancer incidence, and concomitant medications were based on the patients’ self-reported information and may not be sufficiently reliable. The weight data from more than 20 years ago were collected by the hospital staff and are thus unlikely to be associated with the form of inaccuracy that can occur in a recall survey. Of note, we have established a relationship of trust with patients for over 20 years, and I think that patients are submitting accurate data.

The participants in the present study will continue to be followed for clinical outcomes and those who wish to continue treatment with BLP will be allowed to do so. Therefore, continuing the follow-up of this population for approximately 10 more years will allow us to determine whether *Lactobacillus* probiotic preparations have a beneficial effect on both survival and quality of life in the elderly.

## 5. Conclusions

In conclusion, analysis of the data from more than 100 patients who have been treated for more than 20 years with a *Lactobacillus* probiotic preparation suggested that long-term treatment with the product might be effective for suppressing weight loss in the elderly. Careful follow-up of this population, on an ongoing basis, will allow us to evaluate the efficacy of long-term treatment with this product.

## Figures and Tables

**Table 1:** Baseline characteristics of patients who consented to participate.

	BLP Extension		
	No	Yes	
	(No. of patients = 109)	(*n* = 128)	*p*-value
Sex, Male (%)	94 (86.2%)	105 (82.0%)	0.478
At participation in WBB/BLP study			
Age ^1^ (years)	54.3 ± 6.8	54.4 ± 6.0	0.863
Height ^1^ (cm)	164.3 ± 7.4	164.7 ± 7.2	0.700
Weight ^1^ (kg)	64.3 ± 10.5	64.7 ± 9.8	0.797
During WBB/BLP study			
BLP administration			
Yes	44	83	
No	65	45	
WBB administration			
Yes	53	61	
No	56	67	

^1^ Mean ± standard deviation. WBB, wheat bran biscuit; BLP, biolactis powder.

**Table 2:** Distribution of cancer types in patients with and without BLP extension.

Cancer Type	BLP Extension	
	No	Yes
Gastric cancer	10 lesions	10 lesions
Lung cancer	2 lesions	4 lesions
Prostate cancer	4 lesions	3 lesions
Colorectal cancer	2 lesions	2 lesions
Renal/renal pelvis cancer	3 lesions	2 lesions
Bladder cancer	2 lesions	2 lesions
Pancreatic cancer		2 lesions
Liver cancer	1 lesion	1 lesion
Myeloma/lymphoma	1 lesion	0 lesion
Gallbladder cancer	1 lesion	
Breast cancer	1 lesion	1 lesion
Pharyngeal cancer	2 lesions	
Esophageal cancer	1 lesions	

**Table 3:** Changes in weight and BMI over 20 years.

At Participation in WBB/BLP Study	At Questionnaire Survey		Paired *t*-Test
	Approx. 20 Years Later	Over 20 Years	
Weight			
BLP extension (*n* = 124)	64.8 ± 9.8	63.4 ± 10.6	0.011
Non-BLP extension (*n* = 91)	64.2 ± 10.5	61.4 ± 10.4	<0.001
BMI			
BLP extension (*n* = 124)	23.8 ± 2.7	23.5 ± 3.0	0.094
Non-BLP extension (*n* = 91)	23.7 ± 2.6	22.8 ± 2.9	0.001

Mean ± standard deviation. WBB, wheat bran biscuit; BLP, biolactis powder. BMI: body mass index.
